# ATPase-dependent auto-phosphorylation of the open condensin hinge diminishes DNA binding

**DOI:** 10.1098/rsob.140193

**Published:** 2014-12-17

**Authors:** Yuko Akai, Ryuta Kanai, Norihiko Nakazawa, Masahiro Ebe, Chikashi Toyoshima, Mitsuhiro Yanagida

**Affiliations:** 1 Okinawa Institute of Science and Technology Graduate University, Onna-son, Okinawa 904-0495, Japan; 2 Institute of Molecular and Cellular Biosciences, The University of Tokyo, Tokyo 113-0032, Japan

**Keywords:** condensin, auto-phosphorylation, hinge opening, hinge interface, DNA re-annealing, mass spectrometry

## Abstract

Condensin, which contains two structural maintenance of chromosome (SMC) subunits and three regulatory non-SMC subunits, is essential for many chromosomal functions, including mitotic chromosome condensation and segregation. The ATPase domain of the SMC subunit comprises two termini connected by a long helical domain that is interrupted by a central hinge. The role of the ATPase domain has remained elusive. Here we report that the condensin SMC subunit of the fission yeast *Schizosaccharomyces pombe* is phosphorylated in a manner that requires the presence of the intact SMC ATPase Walker motif. Principal phosphorylation sites reside in the conserved, glycine-rich stretch at the hinge interface surrounded by the highly basic DNA-binding patch. Phosphorylation reduces affinity for DNA. Consistently, phosphomimetic mutants produce severe mitotic phenotypes. Structural evidence suggests that prior opening (though slight) of the hinge is necessary for phosphorylation, which is implicated in condensin's dissociation from and its progression along DNA.

## 2. Introduction

**Figure 1.**
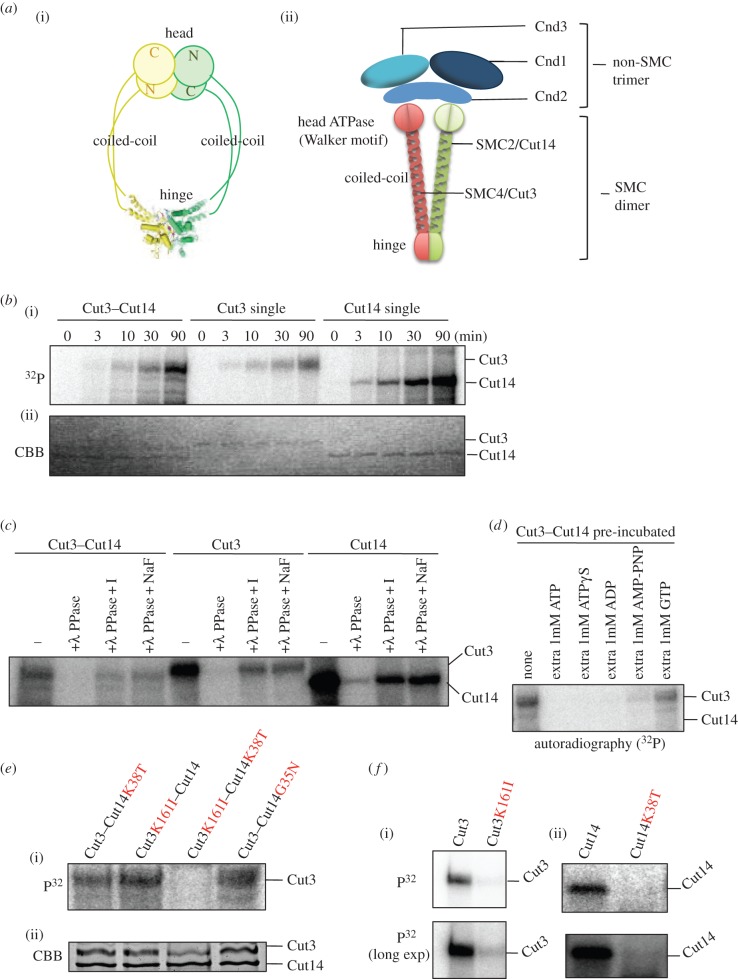
*In vitro* phosphorylation of condensin SMC requires the ATPase domain. (*a*) Illustration of condensin SMC heterodimer (i) and hetero-pentameric holocondensin, consisting of an SMC dimer and a non-SMC trimer (ii). The ATPase domain containing the Walker motif is located in the head domain. The head domain interacts with non-SMC subunits. (*b*) The time course of γP^32^ labelling of Cut3–Cut14, Cut3 and Cut14 subunits in the presence of γP^32^-ATP. See text. (*c*) To confirm that γP^32^-labelled protein was actually phosphorylated, phage λ phosphatase (PPase) was employed to treat proteins after the reaction with γP^32^-ATP. I and NaF are phosphatase inhibitors. (*d*) A large excess of ATP and other nucleotides were added. See text. (*e*) Four Cut3–Cut14 mutant proteins overproduced in *S. pombe* were purified as a complex by affinity chromatography and incubated with radiolabelled γP^32^-ATP (i). The double mutant protein Cut3 K161I–Cut14 K38T-was hardly phosphorylated. CBB-stained protein bands are seen at the bottom (ii). (*f*) Single Cut3 K161I (i) and Cut14 K38T (ii) mutant protein were not γP^32^ phosphorylated, whereas wild-type, single subunits Cut3 and Cut14 were heavily phosphorylated.

The condensin complex contains five distinct subunits, two structural maintenance of chromosome (SMC) subunits, denominated SMC2 and SMC4 (reviewed in [1,2]), and three non-SMC subunits. A number of studies have established that the role of condensin is highly conserved among diverse organisms from bacteria to humans (reviewed in [3]). Condensin controls chromosome condensation, segregation, gene dosage compensation, DNA damage repair and transcriptional control [4–9]. SMC proteins consist of N-terminal and C-terminal domains that fold back onto each other to create an ATPase head domain, connected to a central hinge domain via a long coiled-coil (figure 1*a*(i)). Kimura & Hirano [10] showed that ATP-dependent positive supercoiling of DNA is induced by condensin. Sutani & Yanagida [11] demonstrated that the condensin SMC complex promotes DNA supercoiling and implicated it in chromosome condensation.

The ATPase domain, containing Walker motifs A and B, is composed of two well-separated sequence segments. The hinge domain is reported to mediate dimerization of SMC proteins and binds DNA [12–15]. This activity suggests direct involvement of the hinge domain of SMC proteins in its action on DNA. The trimeric non-SMC subunits are associated with the globular terminal domains of SMC subunits (figure 1*a*(ii)).

In this study, we employed an *in vitro* approach, building upon our previous study, which demonstrated the ability of the condensin SMC heterodimer Cut3–Cut14 to remove single-stranded (ss) DNA-binding protein RPA or Ssb1, which had been bound to the unwound ssDNAs [12]. As the elimination of protein and/or RNA during the re-annealing reaction *per se* did not require ATP, we wondered how ATP interacts with condensin's ATPase domain during the re-annealing reaction. To our great surprise, we discovered that the phosphate groups of ATP bind to multiple sites on Cut3, an SMC4-like subunit, but this apparent ‘auto-phosphorylation’ is greatly diminished when the holocondensin complex, which has ample ATPase activity, is employed. We show that multiple phosphorylation sites are located in the hinge of Cut3/SMC4, and that phosphorylation is abolished in ATPase mutants. We investigated this ATPase-dependent phosphorylation of the hinge in detail; we propose that hinge phosphorylation represents a step in condensin's ATPase cycle, and that it is important for understanding condensin's dissociation from chromosomal DNA. In other words, ATP enables the mobility of condensin along chromosomes by causing it to dissociate from DNA.

## 3. Results

### 3.1. Condensin SMC subunits are γP^32^ phosphorylated by ATP

*Schizosaccharomyces pombe* condensin subunits Cut3/SMC4 and Cut14/SMC2 tagged with haemagglutinin antigen (HA) and polyhistidine (His6) were singly or doubly overproduced in *S. pombe* cells and isolated by affinity chromatography using Ni-beads, as previously described [12]. The resulting homologous and heterologous subunits were incubated with radiolabelled γP^32^-ATP for 0–90 min. After incubation, proteins were run in SDS-PAGE and radioactive protein bands were detected by autoradiography (figure 1*b*(i)). Proteins reisolated after incubation with ATP were stained with Coomassie brilliant blue (CBB) (figure 1*b*(ii)). To our great surprise, γP^32^ labelling occurred intensely in the presence of ATP. γP^32^ labelling of Cut3, Cut14 and Cut3–Cut14 was detected 3 min after the start of incubation, and increased continuously. Cut3 and Cut14 (presumably homodimers if they behaved as bacterial condensin) were strongly radiolabelled, whereas only Cut3 was intensively labelled in the heterodimer.

### 3.2. γP^32^ labelling is removed by λ phosphatase

To examine whether the labelling represented a covalent association of phosphate and protein, we used λ-phosphatase treatment of proteins after incubation with γP^32^-ATP. As shown with autoradiography (figure 1*c*), λ-phosphatase (+λ-PPase) treatment eliminated the labelled band, while its removal was compromised by addition of phosphatase inhibitors. Thus, labelling was due to protein phosphorylation.

### 3.3. Great excess of ADP, but not GTP, inhibits γP^32^ labelling

We then examined whether γP^32^ radiolabelling was inhibited in the presence of a large excess of ATP or other nucleotides. The Cut3–Cut14 complex was first incubated in the presence of 1 mM concentrations of nucleotides (ATP, ATPγS, ADP, AMP-PNP or GTP) for 5 min at room temperature, followed by incubation with radioactive ATP (8 nM) and cold ATP (50 nM) for 30 min at 30°C. All samples were boiled in SDS-PAGE buffer, separated electrophoretically and followed with autoradiography. Prior incubation in excess ATP, ATPγS or ADP abolished ATP γP^32^ labelling, but excess GTP scarcely affected it (figure 1*d*). Some labelling occurred in an excess of AMP-PNP. Inefficient inhibition by excess GTP and AMP-PNP might suggest that efficient inhibition against phosphorylation of SMC subunits via ATP and ADP requires an adenosine diphosphate or triphosphate moiety.

### 3.4. Phosphorylation requires the ATPase domain

To address the question of whether SMC's ATPase domains were required for the *in vitro* phosphorylation described above, we constructed mutant proteins of Cut3 and Cut14 containing amino acid substitutions at essential residues in the N-terminal domains of the ATPase consensus sequence. Glycyl (G) and lysyl (K) residues in the amino terminal ATPase consensus sequence (Walker ATPase motifs, W in the electronic supplementary material, figure S1; see also figure 1*a*(ii) for location in the condensin complex) were altered in the Cut14/SMC2 substitution mutants (G35N, K38T; N, asparagine and T, threonine) and the Cut3/SMC4 mutant (K161I; I, isoleucine). Resulting mutant proteins were overexpressed using plasmids constructed for that purpose, and were isolated by nickel affinity chromatography. Purified mutant proteins were examined for labelling in the presence of γP^32^-ATP. For the Cut3–Cut14 complex, only the double mutant complex Cut3 K161I–Cut14 K38T abolished γP^32^ labelling (figure 1*e*(i); panel (ii) shows CBB-stained bands of purified Cut3 and Cut14 wild-type and mutant proteins). Thus, only one intact ATPase domain was sufficient for γP^32^ labelling in the Cut3–Cut14 complex. For the homodimeric Cut3 containing a K161I substitution, and also for the homodimeric Cut14 containing a K38T mutation (figure 1*f*), γP^32^ labelling was hardly observed. In summary, phospho-labelling requires an intact condensin SMC subunit ATPase domain.

### 3.5. Overproduction of Cut14 G35N produces severe mitotic defects similar to those in ts mutants

**Figure 2.**
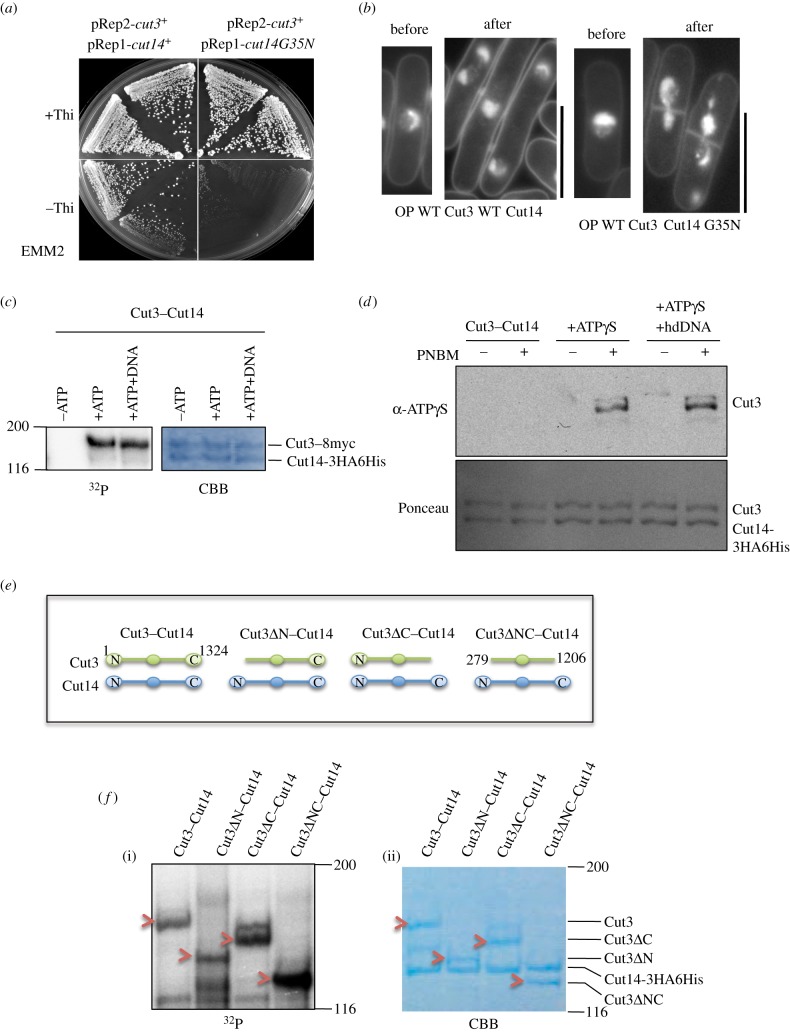
ATPase mutant causes mitotic phenotype, and protein thiophosphorylation can occur in terminally truncated SMC proteins when one of two SMCs is intact. (*a*) Plasmid pREP1 carrying the Cut14 G35N mutant gene under the inducible promoter was used to overexpress it simultaneously with the wild-type *cut3*^+^ gene (plasmid pREP2) in the absence of thiamine (−Thi). See text. (*b*) Cells were stained by DAPI. In liquid culture, a defective phenotype in chromosome condensation and segregation was observed after co-overexpressing (OP) the wild-type Cut3 and the ATPase mutant Cut14 G35N (right panel). See text. Scale bars, 10 µm. (*c*) Cut3–Cut14 complex was incubated with γP^32^-ATP in the presence or the absence of M13 ssDNA. The autoradiography pattern (P^32^) and CBB staining patterns are shown. (*d*) Antibody against thiophosphorylated amino acids (α-ATPγS) was used to detect thiophosphorylated residues of Cut3–Cut14 incubated in the presence of ATPγS, hdDNA and PNBM (the reagent for detecting γS). The Cut3 band containing γS was detected when Cut3–Cut14 was incubated with ATPγS and PNBM, regardless of the presence of hdDNA. Protein bands of Cut3 and Cut14-3HA6His were stained with Ponceau S. (*e*) Wild-type Cut3/SMC4 and Cut14/SMC2, and three single- or double-truncated Cut3 and wild-type Cut14 constructs are shown. (*f*) Wild-type and truncated SMC dimer were incubated with γP^32^-ATP, run in SDS-PAGE, and examined by autoradiography of labelled proteins (i) and CBB staining (ii). Red arrows indicate bands of Cut3 wild-type and mutant truncated proteins in autoradiography and CBB stain.

We examined whether abnormal cellular phenotypes resulted from overproduction by plasmids carrying the ATPase G35N substitution mutant gene. The *cut14 G35N* substitution mutant gene was overexpressed under an inducible promoter, using plasmid pREP1, with co-overexpression of the wild-type *cut3*^+^ gene carried by plasmid pREP2 in the absence of thiamine (−Thi) so that wild-type Cut3 and mutant Cut14 G35N were overproduced, forming the heterodimer and causing a dominant-negative effect (no colony formed; figure 2*a*, right bottom). Under conditions to suppress overexpression (+Thi), *S. pombe* cells carrying the mutant plasmid formed normal colonies (figure 2*a*, right top). Thus, co-overexpression (−Thi) of Cut14 G35N and wild-type Cut3 proteins was strongly inhibitory.

In liquid culture, mitotic chromosome condensation and segregation were defective in cells after co-overexpression of wild-type Cut3 and mutant Cut14 G35N protein (figure 2*b*). The phenotype highly resembled that of the temperature-sensitive (ts) condensin mutants that failed to interact with chromosome DNA [9]. Under expression of both wild-type genes, however, cells showed normal mitosis. Cells were somewhat elongated, suggesting that the G2/M transition might be slightly delayed. Overproduction of ATPase mutant Cut14 G35N protein thus produced the negative-dominant severe mitotic defects similar to those observed in previously isolated condensin mutants, suggesting that condensin was not functional in cells overproducing Cut14 G35N ATPase mutant protein.

### 3.6. γP^32^ labelling is not affected in the presence of ssDNA

To better understand this ATPase domain-dependent γP^32^ labelling, we examined whether the reaction was affected by the presence of ssDNA (single-strand M13 DNA), for which *S. pombe* condensin SMC heterodimer has a very strong affinity [12]. The Cut3–Cut14 complex was incubated in the presence or the absence of M13 ssDNA. No difference was found in the degree of γP^32^ labelling of Cut3 (figure 2*c*). Phosphorylation of condensin Cut3 was hence not affected by the presence of ssDNA.

### 3.7. Cut3–Cut14 complex is thiophosphorylated by ATPγS

ATPγS is useful because proteins can be γ-thiophosphorylated *in vitro*, but phosphatases cannot remove it. It can be detected with a specific antibody [16] (Material and methods). Antibody against ATPγS detected Cut3 protein in the heterodimer after incubation in the presence of ATPγS (+) but not in the absence of ATPγS (−) (figure 2*d*). PNBM is the reagent necessary for immunodetection of ATPγS: thiophosphorylated Cut3 was detected only with the addition of PNBM (*p*-nitrobenzyl mesylate). Ponceau-stained Cut3–Cut14 bands are shown below. Consistent with the above result, the degree of thiophosphorylation was not affected by the presence or the absence of heat-denatured (hd) DNA, suggesting that hdDNA did not affect the degree of phosphorylation.

### 3.8. Phosphorylation sites may reside in the hinge and coiled-coil

We wanted to determine the site(s) of phosphorylation in Cut3/SMC4. To this end, we employed wild-type Cut3, and three truncated mutant proteins, Cut3ΔN, Cut3ΔC and Cut3ΔNC, previously made [17] (schematized in figure 2*e*). They were produced as a complex with wild-type Cut14 by simultaneous overproduction. Truncated complexes were purified, incubated with radiolabelled γP^32^-ATP, run in SDS-PAGE and examined by autoradiography of labelled proteins (P^32^, figure 2*f*(i)). CBB-stained proteins are also shown (figure 2*f*(ii)). All truncated Cut3 bands (red arrowheads) were intensely γP^32^ labelled, suggesting that the hinge and/or coiled-coil domains could be phosphorylated *in vitro*, when Cut14/SMC2 was intact.

### 3.9. Multiple hinge phosphorylation revealed by mass spectrometric analysis

**Figure 3.**
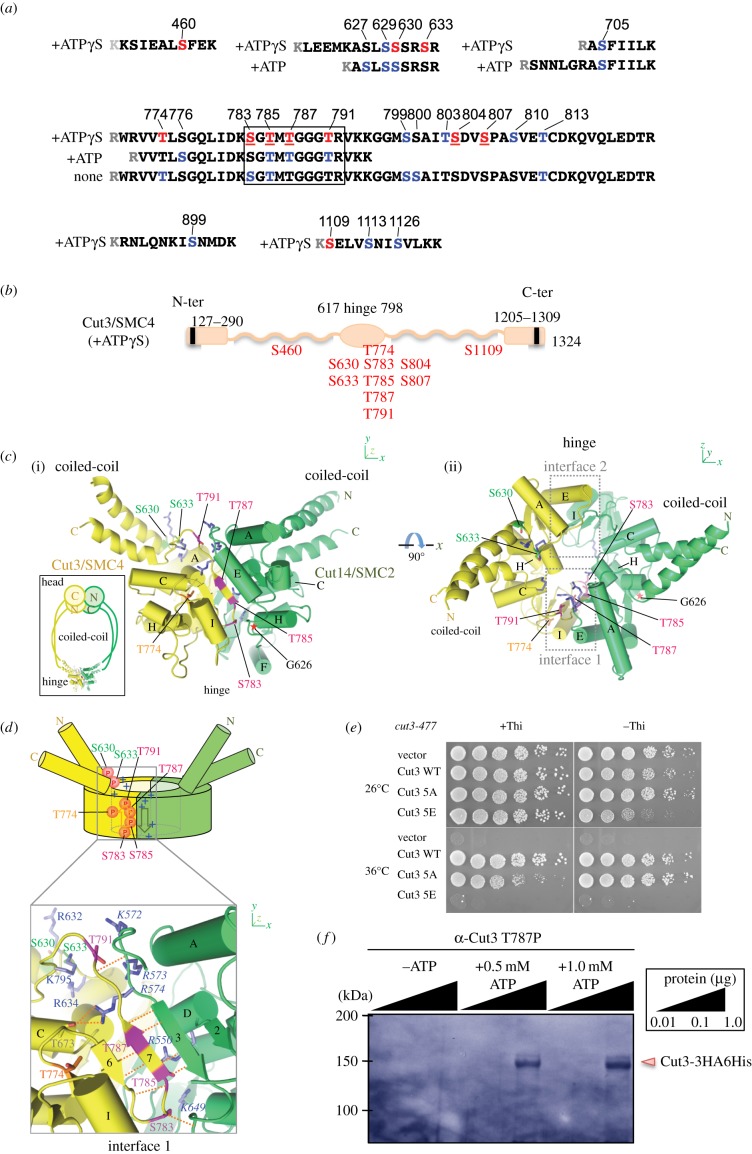
(Thio)Phosphorylation sites of Cut3/SMC4 are located in the G-rich sequence along the hinge dimer interface. (*a*) Purified Cut3–Cut14 was incubated without ATP, or in the presence of ATP or ATPγS, and reisolated for LC-MS analysis. Phospho- or thiophosphopeptides of Cut3 are shown with the amino acid sequence number and the sequences containing phosphorylated (blue) and thiophosphorylated (red) residues (also shown in electronic supplementary material, tables S1 and S2). The majority of residues are located in the hinge (616–798) and hinge-adjacent regions. (*b*) Schematic of Cut3 protein with thiophosphorylated residues (red). The Walker motif sequences shown by the vertical bars (black) are located in the N- and C-termini. (*c*) An expected structure of the heterodimeric hinge of Cut3/SMC4 (yellow) and Cut14/SMC2 (green). Thiophosphorylated residues are located along the interface of the dimer. T785 and T787 are located in a β-strand (β7, see text). The asterisk indicates G626 at the base of helix H that works as a pivot in opening/closing of the Cut14/SMC2 hinge (see Discussion). (*d*) Top panel: locations of the thiophosphorylated residues (red discs labelled P) and the surrounding basic residues (‘+’) in the hinge doughnut (see text). Arrows indicate the β-strands. Bottom: details around interface 1. Thiophosphorylated residues are coloured green (S630 and S633), orange (T774) or magenta (S783, T785, T787 and T791). Surrounding basic residues appear in blue and are labelled with normal letters for Cut3/SMC4 and italics for Cut14/SMC2. Hydrogen bonds are indicated by dotted lines. Basic residues of Cut14/SMC2 are shown in italics. Basic residues of Cut3/SMC4 are also seen. (*e*) *cut3-477* mutant cells were transformed with plasmids carrying no gene (vector), wild-type *cut3*^+^ (WT) and the *cut3* mutant gene containing 5A or 5E substitutions (see text), and resulting transformants were spot tested at 26°C and 36°C in the presence of thiamine (induction is off). (*f*) Immunological detection of the phosphorylated Cut3-T787-P site. Cut3 protein was isolated from cells that overproduced Cut3-3HA6His, incubated in the absence of ATP, or the presence of 0.5 and 1.0 mM ATP, and run on SDS-PAGE. An antibody raised against the peptide KSGTMT^787P^GGGTRYK containing phospho T787 was used for detection.

For mass spectrometric analysis, we purified Cut3ΔNC–Cut14 complex (electronic supplementary material, figure S2), which was incubated in control buffer (no ATP, lane 1) in the presence of ATP (ATP, lane 2) and in the presence of ATPγS (ATPγS, lane 3) at 30°C for 90 min. CBB staining is also shown. Slices of the SDS-PAGE gel were digested with trypsin or lysine-specific Lys-C protease, and resulting digests were analysed by mass spectrometry as described previously [18]. Identified phosphorylated or thiophosphorylated peptides, with ion score values (obtained with Mascot software for Orbitrap) or the number of peptides, respectively, are shown in the electronic supplementary material, tables S1 (trypsin digestion) and S2 (Lys-C digestion). These results are summarized in figure 3*a,b*.

Trypsin-treated Cut3 produced phosphopeptides in the absence (None) or the presence (+ATP) of ATP. The majority of peptides came from the hinge region (aa 616–798; defined by IPR010935) or the hinge-adjacent domain (aa 799–807). The same S or T residues were either phosphorylated (blue character) or thiophosphorylated (red). No modified tyrosine or aspartate residues were detected. Peptides containing the 10-residue, glycine-rich segment 783-S*G*TMT*GGG*TR-792 were found in all three samples, and produced the highest phosphorylation or thiophosphorylation scores in the samples incubated with ATPγS (electronic supplementary material, table S1). We next focused on this highly phosphorylated G-rich peptide in Lys-C digested samples (electronic supplementary material, table S2). In the untreated sample, 16 S*G*TMT*GGG*TR peptides were unmodified, while seven were modified at residues such as S783, T785, T787 and T791, suggesting that Cut3/SMC4 was already phosphorylated in cell extracts. In the presence of ATPγS, 11 peptides were phosphorylated or thiophosphorylated at the same four residues, while no modification was detected in 19 peptides. Thus, the hinge is the major site of thiophosphorylation. It is of interest to note that the G-rich 10-residue stretch is commonly present in SMC proteins, including those of prokaryotic origin [14].

Besides the G-rich stretch, a serine-rich stretch, 627SLSSSRSR634 present in the N-terminus of the hinge was (thio)phosphorylated at positions 630 and 633 (SLSS^630^SRS^633^R). Nine residues (S630, S633, T774, S783, T785, T787, T791, S804, S807) in the hinge or adjacent regions were thiophosphorylated, and four of them (S783, T785, T787 and T791) were the major modified residues (figure 3*b*). Two other residues (S460, S1109) are located in the middle of the coiled-coil. Accordingly, the hinge, and the G-rich stretch particularly, is the principal (thio)phosphorylation target.

### 3.10. Thiophosphorylated Cut3 residues located along the interface with Cut14

Crystal structures for SMC hinges of prokaryotes and eukaryotes have been determined by X-ray crystallography [13,19,20]. To locate (thio)phosphorylated residues identified by mass spectrometry, we constructed an atomic model for the hinge and a part of the coiled-coil of Cut3–Cut14, based on crystal structures from mouse and the hyperthermophilic bacterium *Thermotoga maritima* (figure 3*c*). The hinge has a doughnut-shaped structure in which Cut3/SMC4 and Cut14/SMC2 are related by a pseudo-twofold symmetry (figure 3*d*, top panel). N-terminal and C-terminal ends of each hinge domain are on one face of the doughnut and are connected to α-helices in the coiled-coil connected to the ATPase domain. The doughnut contains two dimer interfaces (1 and 2; figure 3*c*(ii)), each of which is stabilized by an intermolecular β-sheet. Strikingly, the phosphorylated or thiophosphorylated residues of Cut3/SMC4 (S783, T785, T787 and T791) are clustered within or near the β-strand (β indicated by 7 in figure 3*d*, bottom panel) that forms interface 1, and the side chains of the phosphorylated residues on β7 pointing towards the centre of the doughnut, so that these residues seem to be inaccessible to phosphorylation without a considerable conformational change (see also the electronic supplementary material, figure S3). T774, a phosphorylated residue on β also appears inaccessible due to helix I (in Cut3/SMC4) in the dimer interface. Thus, for phosphorylation of these residues to take place, the hinge interface has to open slightly. The remaining (thio)phosphorylated residues, S630 and S633, are exposed, situated in a loop connecting the hinge and coiled-coil region, and may be readily phosphorylated, but actually less readily (thio)phosphorylated.

Interestingly, these (thio)phosphorylated residues are located amidst numerous basic residues, R550, K572, K573, R574 and K649 of Cut14/SMC2 and R632, R634 and K795 of Cut3/SMC4, indicated in the enlarged structure (figure 3*d*, bottom) and also in the doughnut cartoon (figure 3*d*, top, by +), and in the surface electrostatic potential (blue patch, electronic supplementary material, figure S4). These basic residues have been implicated in DNA interaction. Three consecutive basic residues in Cut14/SMC2 (K572–R574) are conserved in SMC2 of budding yeast (Sc SMC2), *Xenopus* (XCAP-E) and human (hCAP-E) (electronic supplementary material, figure S5). Thus, the side of the doughnut connected to the Cut3/SMC4 coiled-coil is highly positively charged, but substantially less so when phosphorylation has occurred. It seems also likely that the orientation of the coiled-coil changes as a result of phosphorylation (of S630 and S633, in particular) and salt bridge formation. These structure changes may be related to DNA's interaction with condensin (see Discussion).

### 3.11. Phosphomimetic mutant gene is impaired in suppressing the ts phenotype of *cut3-477*

We made plasmids carrying the *cut3* substitution mutant genes 5A and 5E at the phosphorylation sites in the G-rich stretch. They were replaced with alanine (5A) or phosphomimetic glutamate (5E) as follows: T774, S783, T785, T787 and T791. Note that these phosphorylatable residues are conserved in eukaryotic condensin SMC4 proteins of *S. pombe*, humans, budding yeast (Sc) and *Xenopus* except for T791, which is not conserved in budding yeast nor in *Xenopus* (electronic supplementary material, figure S6). Plasmid pCut3-5E hardly suppressed the ts phenotype of *cut3-477* (figure 3*e*). However, overproduction plasmid pCut3-5A fully suppressed the ts phenotype (a vector plasmid was the negative control). These results suggested that 5E mutant protein was dysfunctional at 36°C, regardless of whether it was overproduced (–Thi) or suppressed (+Thi). 5E mutant protein may be dominant-inhibitory when overproduced at 26°C (Cut3-477 protein is functional). 5A mutant protein seems to be non-inhibitory when overexpressed (−Thi), but partly impaired at 36°C when suppressed (+Thi).

### 3.12. Immunological detection of ATP addition-dependent Cut3-T787 phosphorylation

We wanted to obtain a different type of result for hinge phosphorylation, so we employed immunochemical methods. To this end, polyclonal antibody, α-Cut3-T787-P, against the phosphopeptide KSGTMT^787P^GGGTRYK was employed for detecting phosphorylation of Cut3 *in vitro* (Material and methods). No band was obtained for Cut3-3HA6His incubated in the absence of ATP using antibody α-Cut3-T787-P (figure 3*f*). In the presence of 0.5 or 1.0 mM ATP, bands corresponding to the position of Cut3-3HA6His protein were clearly detected. Thus, we succeeded in immunochemical detection of Cut3-T787P.

### 3.13. The release of ATP γ-phosphate scarcely occurred for Cut3–Cut14 dimer

**Figure 4.**
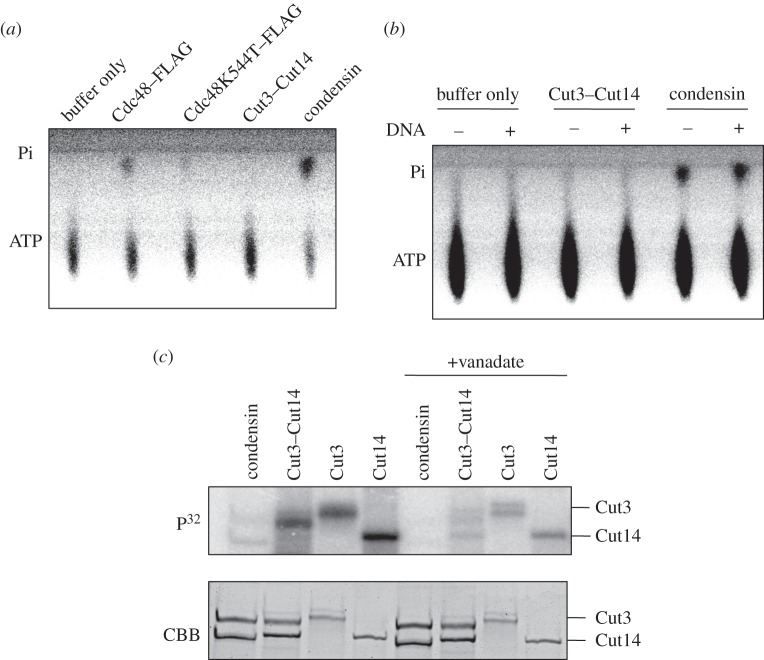
The relationship of ATPase with, and the effect of vanadate on phosphorylation. (*a*) *Schizosaccharomyces pombe* holocondensin employed in this study showed ATPase activity comparable to that of CDC48 ATPase. Activity was detected autoradiographically on thin-layer plates by the release of radiolabelled γP^32^ (Pi) after incubation of condensin with γP^32^-ATP. For positive and negative controls, purified Cdc48/p98 and its ATPase-diminished mutant K544T [21], respectively, were employed. Using the same amount of protein (1 μg), the amount of γP^32^ released by purified condensin was higher than that released by wild-type Cdc48. γP^32^ release was not detected with purified Cut14/SMC2–Cut3/SMC4, however. (*b*) Condensin ATPase activity detected in thin-layer chromatography as the release of radioactive γP^32^ was examined in the presence (+) or the absence (−) of M13 ssDNA (25 ng). Incubation with DNA was for 20 min at 30°C, after which protein was incubated with ATP for 10 min at 30°C. (*c*) (Left four lanes) Purified single SMC (His-tagged Cut3-H or Cut14-H), double SMC (Cut3–Cut14-H) and heteropentameric holocondensin were incubated in the presence of radiolabelled γP^32^-ATP (cold ATP 500 nM, hot ATP 41 nM), and run in 6% SDS-PAGE followed by autoradiography. CBB-stained protein bands are shown below. Single subunits were intensely γP^32^ labelled due to self-phosphorylation. Weak bands were produced for condensin corresponding to Cut3 and Cut14. (Right four lanes) Vanadate (5 mM) is an ATPase inhibitor.

We employed thin-layer chromatography to test whether the purified Cut14–Cut3 complex contained activity that could release γP^32^ from ATP. Release of γP^32^ (Pi) was not detected autoradiographically, in contrast to control holocondensin, which showed ample γP^32^ activity (Pi release), even higher than that of control Cdc48 ATPase (figure 4*a*; Material and methods). Cdc48 K544T [21] is a mutant that possesses diminished ATPase activity and was used as a negative control.

We next investigated whether the presence of DNA might activate the γP^32^ release activity of Cut3–Cut14. M13 ss DNA was mixed with Cut3–Cut14 or condensin. The degree of γP^32^ release was not affected, regardless of the presence (+) or absence (−) of M13 ssDNA (figure 4*b*). These results strongly suggested that the heterodimeric Cut3–Cut14 can phosphorylate itself in the presence of ATP but does not release free γP.

### 3.14. γP^32^ labelling of Cut3 and Cut14 is weak in holocondensin

Holocondensin was then incubated by itself in the presence of γP^32^-ATP. It was isolated using tagged Cut14, as previously described [12]. Both Cut3 and Cut14 in condensin were faintly P^32^ labelled, while control SMC subunits were intensely labelled (HA- and His-tagged proteins) (figure 4*c*, upper panel, left four lanes). The degree of P^32^ labelling was less than 10% of that in Cut3–Cut14, Cut3 and Cut14. As the amount of purified holocondensin obtained was low, modified peptides could not be identified by mass spectrometry.

### 3.15. Inhibition of γP^32^ labelling by vanadate

It was found that 5 mM vanadate inhibited γP^32^ labelling of condensin and SMC subunits (figure 4*c*, right four lanes). Inhibition by vanadate was not complete, as some residual γP^32^ labelling of Cut3 and Cut14 subunits occurred. Vanadate ion is an inhibitor of ATPases, such as Na, K-ATPase, myosin, etc., and is similar to orthophosphate in its charge [22,23].

### 3.16. ATPγS-pretreated wild-type SMC, but not ATPase mutant SMC, fails DNA re-annealing

**Figure 5.**
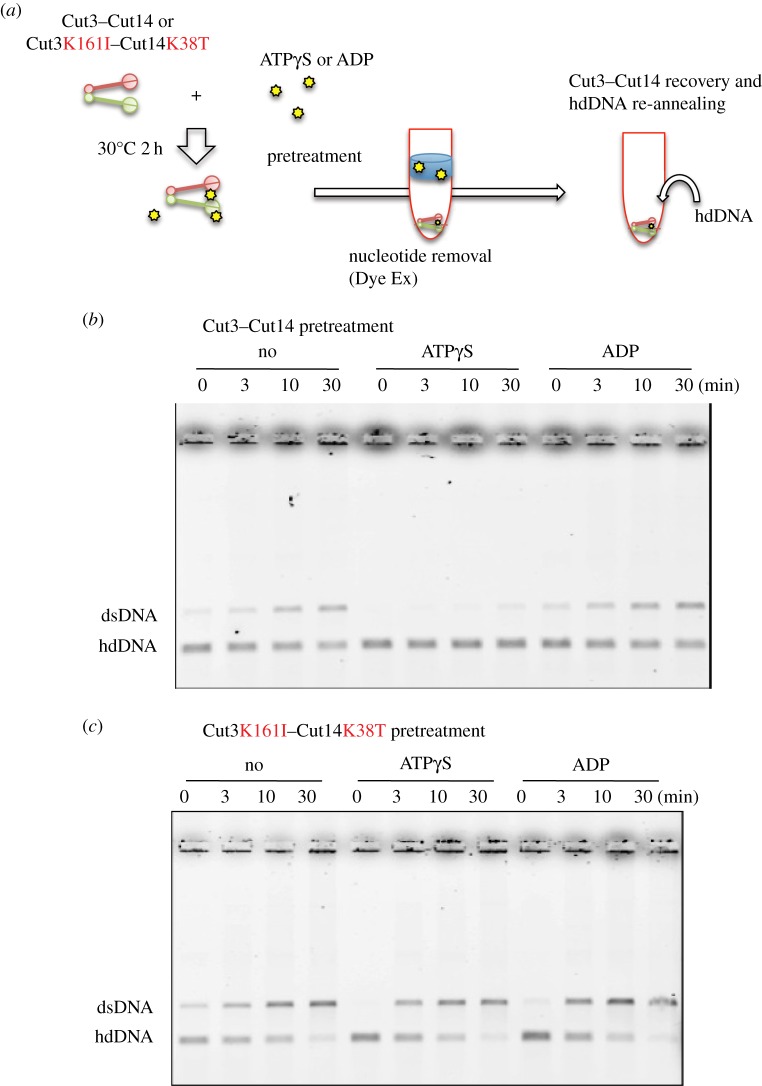
Thiophosphorylated Cut3–Cut14 fails to re-anneal DNA. (*a*) Schematized experimental procedures. See text. (*b*) Purified Cut3–Cut14 (15 nM) was first incubated with 10 mM ATPγS or ADP at 30°C for 2 h, and then treated with Dye Ex (QUIAGEN) to remove free nucleotides. Resulting pretreated Cut3–Cut14 was used to assay DNA re-annealing (30°C for 0–30 min) from hd complementary DNA to ds (double-stranded) DNA. Untreated Cut3–Cut14 (No) was used as the control. After the re-annealing reaction, samples were run in 0.7% agarose gels in the presence of 0.2% SDS. DNA was stained with ethidium bromide. (*c*) The double ATPase mutant protein Cut3 K161I–Cut14 K38T was isolated (figure 1*e*) and used for the same experiment described above.

To obtain information about possible functional changes in Cut3–Cut14 after phosphorylation, we tested whether wild-type and ATPase mutant Cut3–Cut14, pretreated with ATPγS, were able to re-anneal hdDNA into dsDNA (Material and methods, figure 5*a*). Wild-type Cut3–Cut14 and ATPase mutant Cut3K161I–Cut14K38T complex were pre-incubated with ATPγS or ADP for 2 h at 30°C, and then passed through α Dye Ex column (QIAGEN) that eliminated nucleotides. Resulting heterodimer samples were incubated with hdDNA to examine their DNA-re-annealing ability (figure 5*b*). Wild-type Cut3–Cut14 treated with or without ADP could re-anneal hdDNA, whereas wild-type Cut3–Cut14 treated with ATPγS failed to do so, showing that thiophosphorylation rendered Cut3–Cut14 dysfunctional for DNA re-annealing. ADP treatment had no inhibitory effect regarding DNA re-annealing activity, however.

By contrast, the ATPase double mutant Cut3 K161I–Cut14 K38T protein treated with ATPγS could promote re-annealing (figure 5*c*), consistent with the notion that the intact ATPase domain is not required for DNA re-annealing. Actually, the intact domain appeared to be needed for the ATPγS-induced loss of re-annealing ability.

### 3.17. ATP and vanadate restrain DNA–protein and RNA–protein interactions

**Figure 6.**
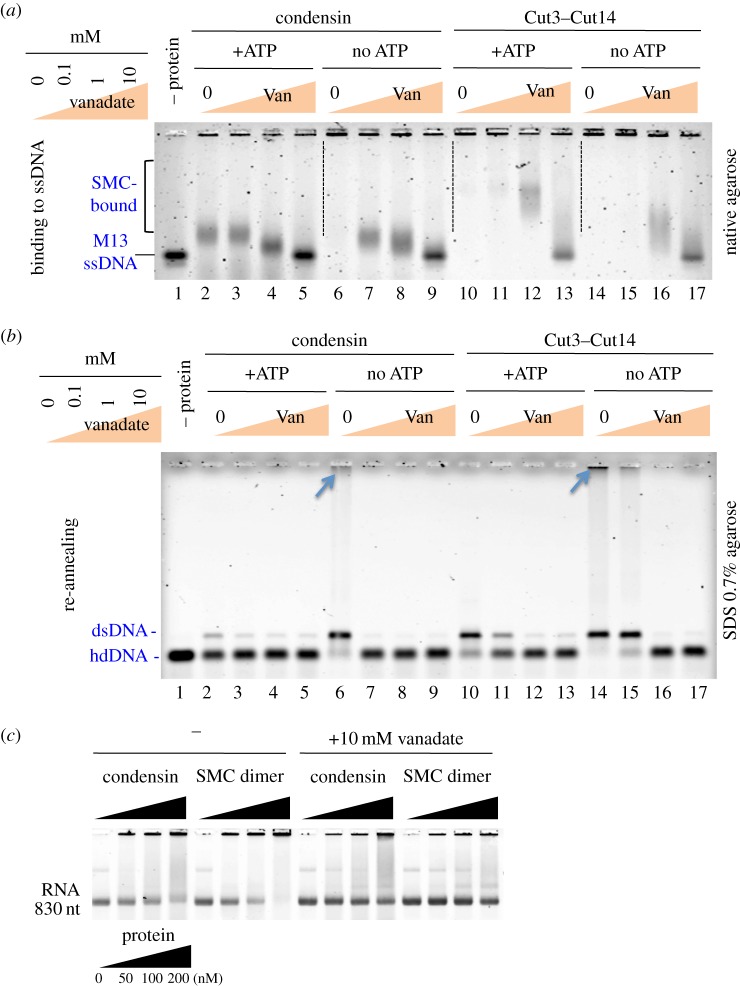
ATP and vanadate diminish DNA–protein interactions. (*a*) *In vitro* binding of condensin and Cut3–Cut14 to M13 ssDNA in the presence or the absence of ATP (10 mM) and vanadate (Van 0, 0.1, 1 and 10 mM) was assayed in a native gel and stained with SYBR Gold. The concentrations of SMC dimer and holocondensin, respectively, are the same, 50 nM. (*b*) The DNA re-annealing reaction in the presence or the absence of ATP and vanadate as described in (*a*) was assayed by the band shift of hd complementary DNA to dsDNA. DNA was run in an agarose gel in the presence of SDS and stained with ethidium bromide. A large DNA aggregate formed in the absence of ATP (indicated by the arrows at the top). (*c*) RNA binding of condensin and Cut3–Cut14 heterodimer was inhibited by vanadate. 830 nt RNA ([12]; 8 μM) was mixed with increasing amounts of condensin or Cut3–Cut14 dimer (0–200 nM) that had been previously incubated in the presence or the absence of 10 mM vanadate for 10 min at 30°C. Mixtures were electrophoresed in 4% Nusieve agarose gels in the absence of SDS. RNAs were visualized by staining the gel with SYBR Gold. See text.

To monitor how ATP and vanadate affect interactions between DNA and SMC or between DNA and condensin, mixtures of DNA–condensin or DNA–SMC were incubated with increasing concentrations of vanadate in the presence or the absence of ATP. Vanadate is a known phosphate analogue, which presumably acts as an inhibitor of the ATPase of condensin in the absence of ATP as vanadate should tightly bind to the space where γ-phosphate of ATP should be associated. Samples were then run in a native agarose gel and stained with SYBR Gold. M13 ssDNA, showing a single, intense band in the absence of protein (figure 6*a*, lane 1), became very broadly diffuse after being mixed with condensin (lane 6) or Cut3–Cut14 (lane 14) in the absence of ATP (no ATP).

In the presence of ATP, the broad condensin bands shifted (figure 6*a*, lane 2), but the protein–DNA complex band was still very diffuse for Cut3–Cut14 (lane 10). Addition of increasing concentrations of vanadate (0, 0.1, 1 and 10 mM) revealed sharpened bands (lanes 3–5, 7–9 for condensin) and (lanes 12–13, 16–17 for Cut3–Cut14). The interaction between M13 ssDNA and holocondensin was thus weaker than that between ssDNA and Cut3–Cut14 sub-complex. Increasing concentrations of vanadate decreased DNA–protein interactions, resulting in dissociation of DNA from protein. The above results strongly suggested that interactions between ssDNA and condensin or Cut3–Cut14 are restrained by ATP and more efficiently by ATPγS or vanadate.

We then examined the effect of ATP and vanadate on DNA re-annealing promoted by condensin and the Cut3–Cut14 complex [12]. Protein–DNA mixtures were run in SDS, 0.7% agarose (Material and methods; figure 6 legend) and stained with ethidium bromide. The hd complementary DNA band is shown in figure 6*b*, lane 1 (in the absence (–) of protein). In the absence of ATP, DNA re-annealing occurred to form the band of dsDNA (lanes 6 and 14). Large DNA aggregates that stacked on the top of gel also formed (indicated by the arrows) [12]. In the presence of ATP (10 mM), DNA annealing by condensin was greatly diminished (lane 2) and also moderately diminished for Cut3–Cut14 (lane 10). Note that the large aggregates disappeared in the presence of ATP. Thus, the inhibitory effect of ATP on DNA annealing was clear, even for Cut3–Cut14. With increased addition of vanadate, DNA annealing scarcely occurred. Vanadate treatment thus resembled ATPγS treatment.

Condensin and SMC dimer bind to 830 nt long RNA [12], and the binding can be seen on a 4% Nusieve agarose gel in the absence of SDS. RNA was visualized with SYBR Gold. When 200 nM condensin or SMC dimer protein was mixed with 8 μM RNA (70 μg) and incubated at 30°C for 10 min, a large fraction of RNA diffused or remained at the top of the gel (figure 6*c*, left panels). However, if 10 mM vanadate was pre-incubated with condensin or SMC dimer at room temperature for 10 min, RNA–protein binding was significantly diminished (figure 6*c*, right panels). Vanadate thus diminished the interaction of condensin Cut3–Cut14 dimer with RNA. Taken together, vanadate and ATPγS interfere with the interaction between condensin and DNA, probably through irreversible association with phosphorylation targets on condensin. Vanadate-treated SMC subunits behaved like thiophosphorylated SMC, which failed to interact with DNA and possibly also RNA. Condensin might act to remove ssDNA-binding proteins such as Ssb1, or transcripts, prior to mitotic chromosome segregation, as unwound chromosome DNAs are bound to numerous RNAs or proteins, causing extended and diffuse chromatin in interphase cells [24].

## 4. Discussion

In the present *in vitro* experiments, we demonstrated that the hinge of *S. pombe* condensin was intensely phosphorylated by γP^32^, provided that its ATPase Walker motif in the head domain is intact. Abolishment of phosphorylation by amino acid substitution mutations introduced into the Walker ATPase motif of both SMC subunits excludes the possibility of unknown protein kinase contaminations in our protein fractions. This is the first evidence that the condensin ATPase domain intensely phosphorylates the hinge, probably through direct interaction at phosphorylated sites. We suppose that phosphorylation also occurs *in vivo*, as mass spectrometric analysis indicated that the same phosphorylated peptides of the Cut3/SMC4 hinge were present in cell extracts that had not been treated with ATP (electronic supplementary material, tables S1 and S2, None). This phosphorylation may represent an intermediate step in condensin's ATPase cycle. ATPase and phosphorylation activities showed an inverse relationship for condensin and the SMC dimer. Non-SMC subunits (Cnd1, Cnd2 and Cnd3) might be required for the putative dephosphorylation step. The reason that holocondensin was only weakly phosphorylated may be that the ATPase cycle is fully active so that γP is continuously released. SMC subunits alone, however, lack γP-releasing activity, so that phosphorylated SMC subunits accumulated significantly.

Phosphorylation seemed to be due to self-catalytic activity of condensin subunits. To the best of our knowledge, such auto-phosphorylation has not been reported for any SMC proteins. P-type ATPases are known to be auto-phosphorylated, however [25,26]. Archaeon P-type ATPase is auto-phosphorylated in the presence of ATP and is inhibited by vanadate. In P-type transport ATPases, such as the calcium and Na^+^–K^+^ pumps, the phosphoryl γP of ATP is transferred to the aspartate residue as an intermediate, and then γP is eliminated (dephosphorylation) from the protein, resulting in a change in protein conformation, followed by ion transport within the molecule. In P-type ATPases, one unique aspartate residue is auto-phosphorylated and subsequently the phosphorylation site is dephosphorylated for completion of the ATPase cycle. Condensin SMC phosphorylation described in this study displays multiple modifications of serine and threonine residues. Thus, auto-phosphorylation of condensin Cut3/SMC4 differs from the canonical auto-phosphorylation of P-type ATPases.

**Figure 7.**
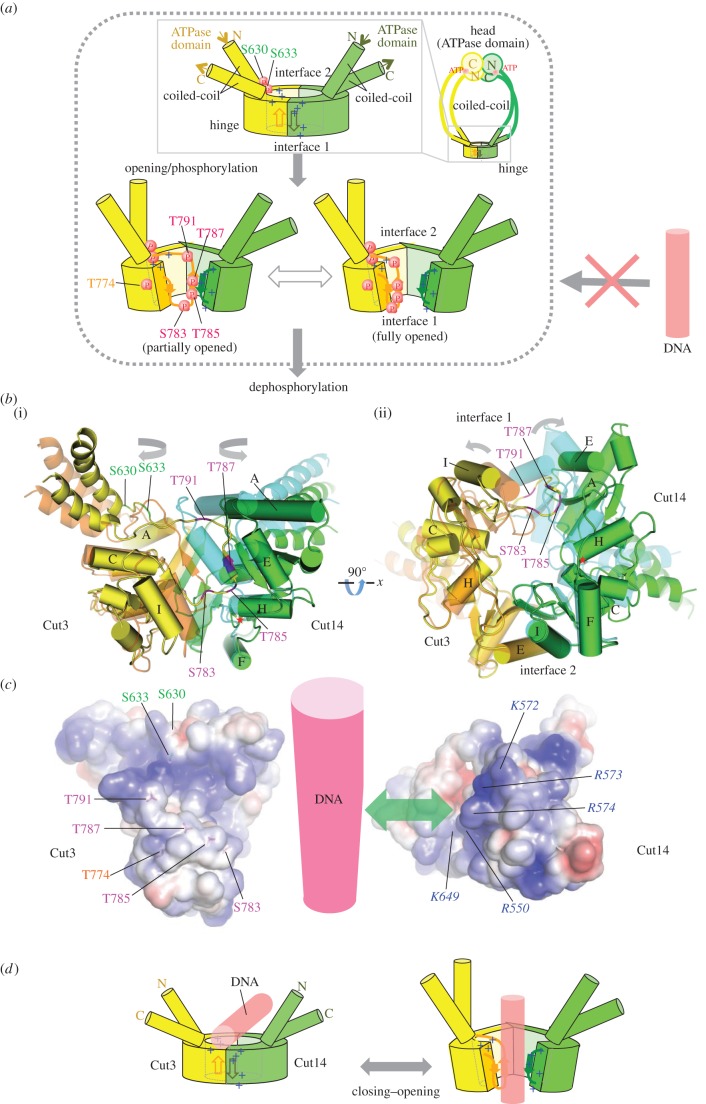
For phosphorylation, opening of the hinge may be required. (*a*) Schematized opening, (auto)phosphorylation and dephosphorylation of the hinge. Schematized whole condensin shown at right top. The phosphorylated hinge is presumed to be unable to bind ssDNA (red DNA rod with the crossed arrow). This hypothesis is based on two crystal structures (closed and opened) of the hinge [13,19,20]. Hinges of Cut3/SMC4 (in yellow) and Cut14/SMC2 (in green) are illustrated, showing locations of thiophosphorylated residues. (*b*) Closed and opened hinges. The closed hinge is coloured orange (Cut3/SMC4) and cyan (Cut14/SMC2). The open hinge is coloured yellow (Cut3/SMC4) and green (Cut14/SMC2). (*c*) Electrostatic surface potential (blue, positive charge; magenta, negative charge) calculated for Cut3 (left) and Cut14 (right) is shown. Characters of basic residues (italic for Cut14) are indicated in blue, with phosphorylated residues in magenta (S783, T785, T787, T791), orange (T774) or green (S630, S633). (*d*) See text for the hypothesis for hinge's association with or dissociation from DNA.

The G-rich sequence 783-*S*G*T*M*T*GGG*T*R-792 of Cut3/SMC4 hinge contains four phosphorylation sites, S783, T785, T787 and T791. In addition, S630, S633 and T774 are phosphorylated. They are identified in an atomic model derived from crystal structures of prokaryotic and eukaryotic SMC proteins [13,19,20] and illustrated in the top view of the Cut3–Cut14 SMC dimer (electronic supplementary material, figure S3, see also figure 3*c,d*). They are clustered in a region near the junction with the coiled-coil and around the interface with Cut14/SMC2. Phosphorylated residues S783, T785, T787 and T791 of Cut3/SMC4 are located on one side of the doughnut surrounded by basic residues (K572, R573, R574 and others) of Cut14/SMC2 that form a highly positively charged patch (blue patch in electronic supplementary material, figure S4) suitable for interaction with DNA. It is easy to see that the phosphorylated hinge will no longer be able to bind to DNA. However, how phosphorylation of these residues is made possible at all remains obscure, as the side chains appear to be inaccessible in the atomic model, in particular for the residues on the Cut3/SMC4 β-strand (β7) that forms an interface with Cut14/SMC2 (figure 3*d*, bottom). Thus, opening of the doughnut structure to remove the steric hindrance seems to be an absolute structural requirement for phosphorylation to occur. Note that hinge opening (figure 7*a*), mentioned throughout this paper, is slight, and is not analogous to the dissociation or splitting proposed for the cohesin hinge [27,28]. If dephosphorylation occurs by the completion of the ATPase cycle, the hinge is again ready for association with ssDNA (or RNA).

There are two crystal structures showing partially opened hinges of mouse SMC2/SMC4 and *T. maritima* SMC homodimers [13,19,20]*.* They exhibit several interesting structural features that lead us to propose a plausible scenario for the phosphorylation as described above. Hypothetical partial opening is realized by changing the orientation of helix H in Cut14/SMC2, which results in a 16 Å movement of helix E (figure 7*b*; the closed structure of Cut14/SMC2 is shown by semi-transparent light blue). The residue at the pivot of helix H is Gly626 (red asterisk in the figure), which is well conserved in SMC2, but not in SMC4. In the atomic models, the local structure around helix H of Cut14/SMC2 appears rather strained in the closed doughnut, but the strain is relieved in the opened structure, forming more hydrogen bonds. In the opened model generated based on the crystal structure of the opened SMC hinge from *T. maritima* (depicted in figure 7*a* bottom left [13]), the intermolecular β-sheet formed by β7 of Cut3/SMC4 and β3 of Cut14/SMC2 is maintained, but β7 is widely separated from β6 within Cut3/SMC4 (figure 7*a*, bottom left, and see also electronic supplementary material, figure S7). In fact, the number of hydrogen bonds that bridge adjacent β-strands varies, and β6 of Cut3/SMC4 is poorly stabilized. On the other side of the doughnut, interface 2 is stabilized by several hydrogen bonds, including salt bridges between R697 (SMC4) and D654/D656 (SMC2), making interface 2 unlikely to open. Furthermore, the coiled-coils may affect the opening and closing of the doughnut structure, as the hinge in these partially opened crystal structures is devoid of associated coiled-coils. These structural features involving coiled-coils suggest that a fraction of the hinge doughnut may be opened even in unphosphorylated states and that phosphorylation of the Ser residues (Ser630 and Ser633) near the junction between the doughnut and the coiled-coil might open the doughnut by changing the orientation of the coiled-coil. These possibilities may be relevant for initiating a structural change in the hinge required for opening. It is conceivable that infrequent phosphorylation of other residues (S460, S804, S807 and S1109, electronic supplementary material, table S1) in the coiled-coil assists the ATPase domain in accessing the hinge.

Structural evidence that opening of the hinge has to precede phosphorylation along the interface 1 (figure 3*c*(ii)), which is dependent on the ATPase domain, raises another argument that phosphorylation that requires hinge opening is physiological rather than artifactual. It may be noteworthy that the hinge mutation for *cut14-Y1* (L543S) lost DNA-binding activity at high temperature [12]. L543 is located far from the positively charged patch on the hinge doughnut surface. Indeed, L543 might work as a mechanical couple that coordinates movements of different parts of the hinge in opening and closing of the doughnut by making contact with F625, the residue next to G626 that works as the pivot (figure 7*b*(ii) and electronic supplementary material, figure S7). Hence, the L543S mutant would fail to open/close the hinge so that the L543S mutant condensin fails to associate with ssDNA. Furthermore, all mutations that rescue the impairment in DNA binding with the L543S mutant are all mapped within the Cut14/SMC2 hinge domain and introduce bulkier residues than the native ones [12]. These observations indicate that opening and closing of the doughnut is a pre-requisite for binding to or dissociating from DNA and would occur under physiological conditions, thereby permitting multiple phosphorylations of residues near the dimer interface.

Figure 7*c* shows electrostatic surface potentials (blue, plus charge; magenta, minus charge) of Cut3/SMC4 (left) and Cut14/SMC2 (right) calculated for the non-phosphorylated subunits. The positively charged patch is clearly seen in the Cut14/SMC2 subunit. This basic patch has been reported in SMCs from bacteria to mammals [14,29] and is thought to comprise the site for DNA interaction. Phosphate groups of DNA (presumably ssDNA) in figure 7 (red column may directly interact with this basic patch in Cut14/SMC2. Phosphorylatable residues in Cut3/SMC4 located alongside the interface are situated close to basic residues in the basic patch of Cut14/SMC2.

We suggest a simple model for the condensin SMC association with and dissociation from DNA (figure 7*d*). Upon association of ssDNA with the central basic patch of the hinge doughnut, the condensin hinge may be slightly open. Note that the full dissociative opening postulated for cohesin, embracing DNA [27,28], is not needed here. The negatively charged phosphate groups of ssDNA may play an essential role for stabilizing hinge opening. Phosphate groups of ssDNA may initially interact with the highly conserved K572, R573 and R574 residues. There may exist two means of dissociation from DNA. dsDNA shows much less affinity to SMC dimer than ssDNA [12] so that dsDNA, formed by re-annealing, may be dissociated from the hinge. Alternatively, ATPase domain-dependent phosphorylation of the hinge might occur initially at the sites other than the G-rich sequence and cause the slight hinge opening followed by phosphorylation of the G-rich sequence, causing dissociation of DNA, as described above. The action of non-SMC subunits might be implicated in eliminating phosphate groups from Cut3/SMC4 so that the hinge dimer is again capable of binding DNA. Another possibility is that the binding of the non-SMC subunits to the SMC head domains restricts hinge opening, which in turn reduces the efficiency of auto-phosphorylation at the hinge. ATP might be used for temporal conformation change that enables buried residues to be phosphorylated. In the absence of the ATPase domain, association and dissociation might occur if initial ssDNA is complementary and converted into dsDNA by re-annealing. If no complementary DNA strand is present, condensin and ssDNA are blocked at the stage of association and form large aggregates [12,17]. Further study is required to definitively examine hypotheses for the ATPase cycle of condensin proposed here.

The G-rich sequence is highly conserved in eukaryotes and also in homodimeric bacterial condensin. Hirano *et al.* [30] and Hirano & Hirano [14] showed that the G-residues in the hinge of bacterial condensin homodimer are required for hinge dimerization. Further, Ala-substitution (AAAA) mutations strengthen the interaction with DNA, whereas Asp-substitution (DDDD) mutations appeared to reduce DNA binding. Bacterial condensin showed ATPase activity upon DNA binding, but *S. pombe* condensin failed to show an effect of DNA binding on ATPase and ATP-dependent phosphorylation. This study indicates that the ATPase domain directly interacts with the hinge G-rich sequence upon hinge opening and temporarily phosphorylates to ensure dissociation of condensin from DNA. The central hinge and the terminal ATPase domain of SMC are separated by a long coiled-coil so that phosphorylation might require their direct interaction. Our previous atomic force microscopy (AFM) study produced clear images of condensin SMC complex as a bent or kinked structure that appeared to bring the hinge into direct contact with the head of the ATPase domain (illustrated in fig. 1e and fig. 2e in [31]). Alternatively, phosphorylation may occur in trans by the ATPase of different condensin complex. Some AFM images also showed that the hinge directly interacts with DNA. A paired condensin structure forming the ring was also found, suggesting that DNA re-annealing reactions that involve two ssDNAs might require the paired complex**.**

## 5. Material and methods

### 5.1. Protein purification and plasmids

Simultaneous overproduction and purification of condensin SMC subunits were performed as described previously [11]. To purify holocondensin and SMC subunits, plasmids were used as described [17]. The *S. pombe* condensin SMC genes, *cut3*^+^ and *cut14*^+^, were subcloned into pREP1 (*LEU2* marker) and pREP2 (*ura4*^+^ marker), respectively, and overexpressed under the *nmt1* promoter in the absence of thiamine. All plasmid constructs were verified by sequencing. Cut3 mutant constructs were tagged with 8Myc at the C-termini, whereas Cut14 constructs were tagged with 3HA-His_6_. For production of heteropentameric holocondensin in fission yeast, plasmids were used as described previously [31]. The *cut3*^+^ and *cut14*^+^ genes, both of which are fused to the *nmt1* promoter, were inserted in tandem into the pUC119 vector with the *LEU2* marker gene and the *ars1* sequence (plasmid pET115). Non-SMC regulatory subunit genes *cnd1*^+^, *cnd2*^+^ and *cnd3*^+^ were also fused to the *nmt1* promoter and similarly cloned into pUC119 with the *ura4*^+^ marker and the *ars1* (plasmid pET110). The *cnd2*^+^ gene was C-terminally tagged with HA and six-histidine sequences for affinity purification.

### 5.2. DNA re-annealing assay

The DNA re-annealing assay was carried out as described previously [11,17]. For the reaction containing ATP, buffer A (20 mM Tris–HCl pH 7.5, 50 mM NaCl, 10 mM MgCl_2_, 1 mM dithiothreitol (DTT), 10% glycerol) containing various concentrations of ATP was used. For the reaction containing vanadate [23], buffer A containing various concentrations of vanadate was used.

### 5.3. ATPase assay

ATPase activity of condensin or SMC dimer proteins was assayed by measuring released ^32^Pi using the radiolabelled [γ-^32^P]ATP (Amersham). Reactions were performed by the procedures described previously [21]. Purified protein (0.5–1 μg) was added to a final volume (20 μl) of the assay mixture that contained 20 mM HEPES, 50 mM KCl, 1 mM DTT, 5 mM MgCl_2_, 200 μM ATP and 20 nM [γ-^32^P]ATP. The reaction was stopped by addition of 1% SDS. Aliquots of each reaction were spotted onto a polyethyleneamide-TLC (thin-layer chromatography) plate (Macherey-Nagel). ATP and Pi were separated by chromatography in 1 M formic acid and 0.5 M LiCl. Products were analysed in an FLA7000 (GE Healthcare). The Tris buffer used in figure 4*a,b* contained 20 mM Tris–HCl (pH 7.5), 1 mM DTT, 10% glycerol, 100 mM NaCl, 2 mM MgCl_2_, cold ATP (2 mM) and labelled ATP (16.6 μM) with or without M13 ssDNA (25 ng/1.6 μM).

### 5.4. *In vitro* auto-phosphorylation reactions followed by SDS-PAGE and autoradiography

His-tag constructs have been previously described [12]. These His-tag proteins were isolated by affinity chromatography, using a poly-histidine tag. Radioactive [γ-P^32^P]ATP (83 nM) and cold ATP (50 μM) were mixed and incubated with affinity purified protein preparations (each 100 nM) for 0–90 min at 30°C. Mixtures were boiled in SDS-PAGE sample buffer and run in 6% polyacrylamide gels, followed by autoradiography. After SDS-PAGE, proteins were stained with CBB. Purified proteins were incubated with ATP mix (41 nM radiolabelled [γ-^32^P]ATP, 500 nM unlabelled ATP) in buffer A (20 mM Tris–HCl pH 7.5, 50 mM NaCl, 10 mM MgCl_2_, 1 mM DTT, 10% glycerol) for 30 min–1.5 h at 30°C. Radiolabelled proteins were separated by SDS-PAGE. Products were analysed in an FLA7000 (GE Healthcare).

### 5.5. *In vitro* thiophosphorylation reactions followed by antibody detection

To detect thiophosphate bound to proteins, we used an anti-thiophosphate ester-specific antibody (EPITOMICS) as described [16]. For thiophosphorylation measurements, proteins were incubated with 10 mM ATPγS. Proteins were alkylated with 2.5 mM I-nitrobenzyl mesylate (PNBM) for 1–2 h at room temperature, and the products were analysed by western blotting. For western blotting, antibodies diluted 1 : 5000 in TBS (pH 8.0) containing 0.5% Tween 20 (TBST) and 5% milk were used. Blots were rocked overnight at 4°C, incubated with anti-rabbit HRP antibody (1 : 5000, GE Healthcare) and imaged.

### 5.6. Identification of phosphorylation sites by mass spectrometry

Purified proteins (2 µg) were incubated with 10 mM ATPγS in buffer A for 1.5 h at 30°C. After boiling in SDS sample buffer, samples were separated by SDS-PAGE (6% gel). After in-gel digestion with modified trypsin (Roche), the resulting peptides were analysed by online liquid chromatography–tandem mass spectrometry on a Finnigan LCQ Advantage (Thermo Fisher), as previously described [18,32]. All tandem mass spectra were searched against the *S. pombe* non-redundant protein database, including common contaminants such as trypsin and keratin, using Mascot (Matrix Science, London, UK).

### 5.7. Generating atomic models of opened and closed hinges for *Schizosaccharomyces pombe* SMC

For homology modelling, the sequences of *S. pombe* Cut3 and Cut14 were aligned with those of SMC4 or SMC2 from mammals and SMC from *T. maritima*. Then, atomic models of opened and closed SMC hinge from *S. pombe* were generated from the crystal structures of SMC hinges from *T. maritima* (PDB codes 1GXJ and 1GXL, [13]) and mouse (PDB codes 2WD5 and 3L51, [20,29]) based on a sequence alignment using Modeller [33].

## Supplementary Material

Supplemental Figures and Tables
