# Supplementary material for: ATPase-dependent auto-phosphorylation of the open condensin hinge diminishes DNA binding
Source: Open Biol. 2014 Dec 17;4(12):140193. doi: 10.1098/rsob.140193 (PMC4281712; doi:10.1098/rsob.140193)
Supplement: Supplemental Figures and Tables [file rsob140193supp1.pdf]

Supplemental Figure s1

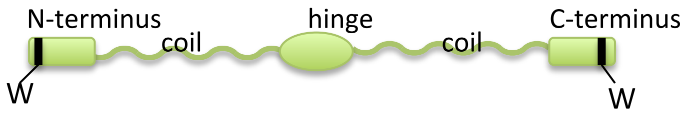

Cut14:GLNG^{35}SGK^{38}S

Cut3:GPNGSGK^{161}S

Consensus :GxxGxGKT/S

Supplemental Figure s1.
Schematic representation of the N- and C-terminal locations of Walker (indicated by the bar and W) ATPase motifs, respectively, in the Cut3/SMC4 and Cut14/SMC2 subunits. Locations of the coiled coil and the hinge are also depicted. Essential ATPase residues (G35, K38 for Cut14 and K161 for Cut3 in red) were used for mutant analysis in this study.

Supplemental Figure s2

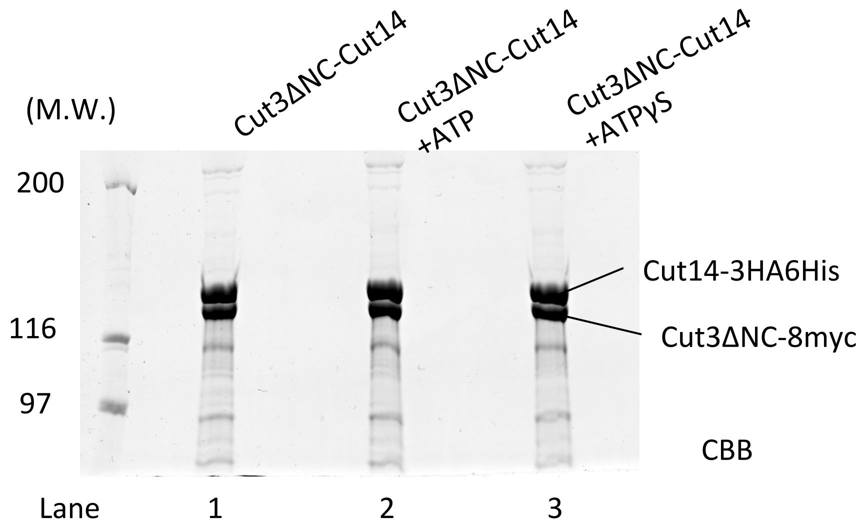

Supplemental Figure s2.
For mass spectrometric analysis, we employed purified Cut3ΔNC-Cut14 complex that was incubated in the absence of ATP (lane 1), the presence of ATP (lane 2), and ATPγS (lane 3) at 30°C for 90 min. Resulting protein preparations were used for LC-MS spectrometry after digestion by trypsin or Lys-C proteinase.

Supplemental Figure s3

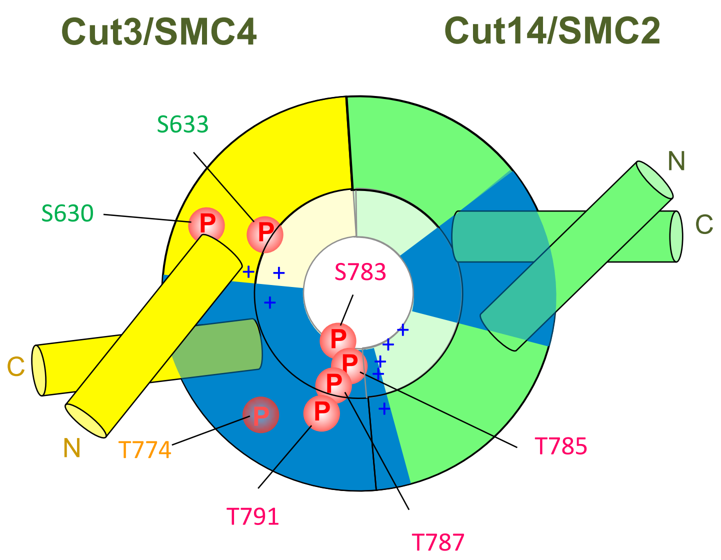

Supplemental Figure s3.
Schematic representation of the hinge and a part of the coiled-coil. Red disks labelled P and '+', respectively, indicate phosphorylated and basic residues. Cut3/SMC4 is colored in yellow and Cut14/SMC2 in green. The strongly positively charged area is shaded blue. The rod is a part of the coiled-coil.

Supplemental Figure s4

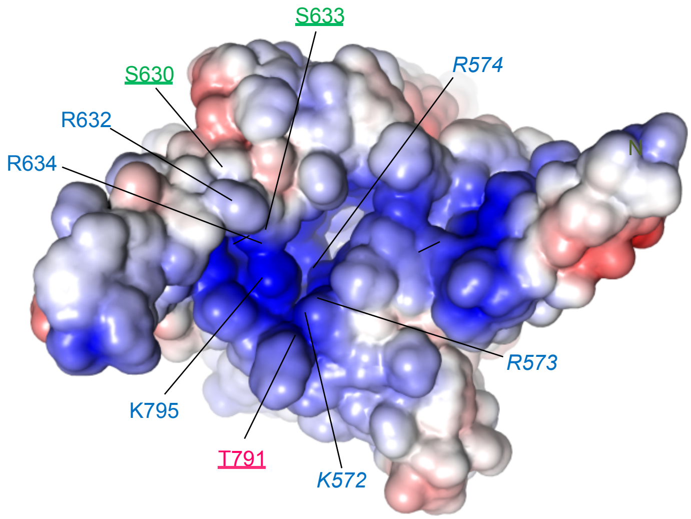

Supplemental Figure s4.
Surface electrostatic potential of the hinge of Cut14/SMC2. Phosphorylated residues are underlined. Basic residues of Cut3/SMC2 are indicated in italics. K572, R573, and R574 are highly conserved in SMC2.

Supplemental Figure s5

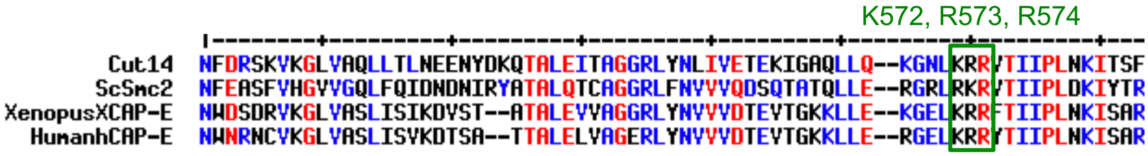

Supplemental Figure s5.
Conserved basic residues in SMC2/Cut14 hinge.

Supplemental Figure s6

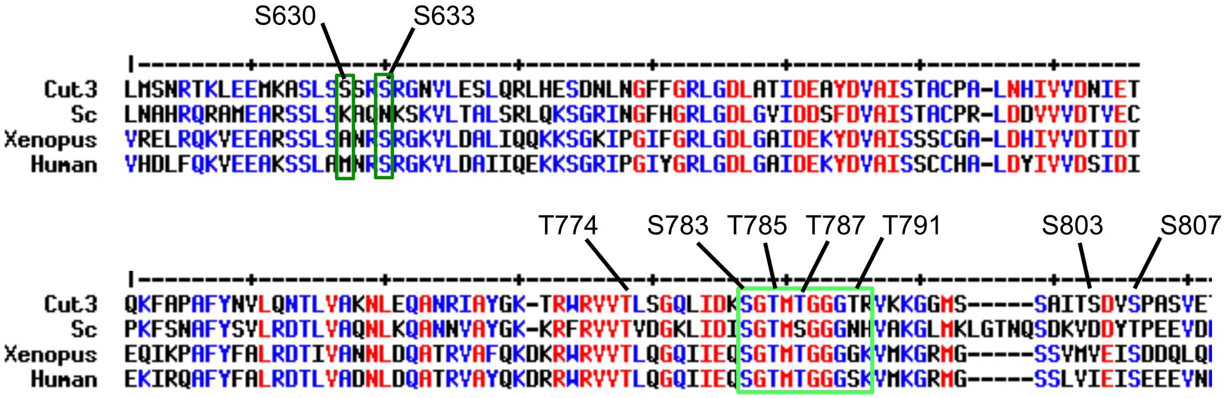

Sc: budding yeast

Supplemental Figure s6.
Conserved hinge amino acid sequences of Cut3, Sc SMC4, *Xenopus*, and human SMC4 homologs. (Thio)phosphorylated residues are indicated by the green frame in Cut3/SMC4 hinge. Sc, budding yeast.

Supplemental Figure s7

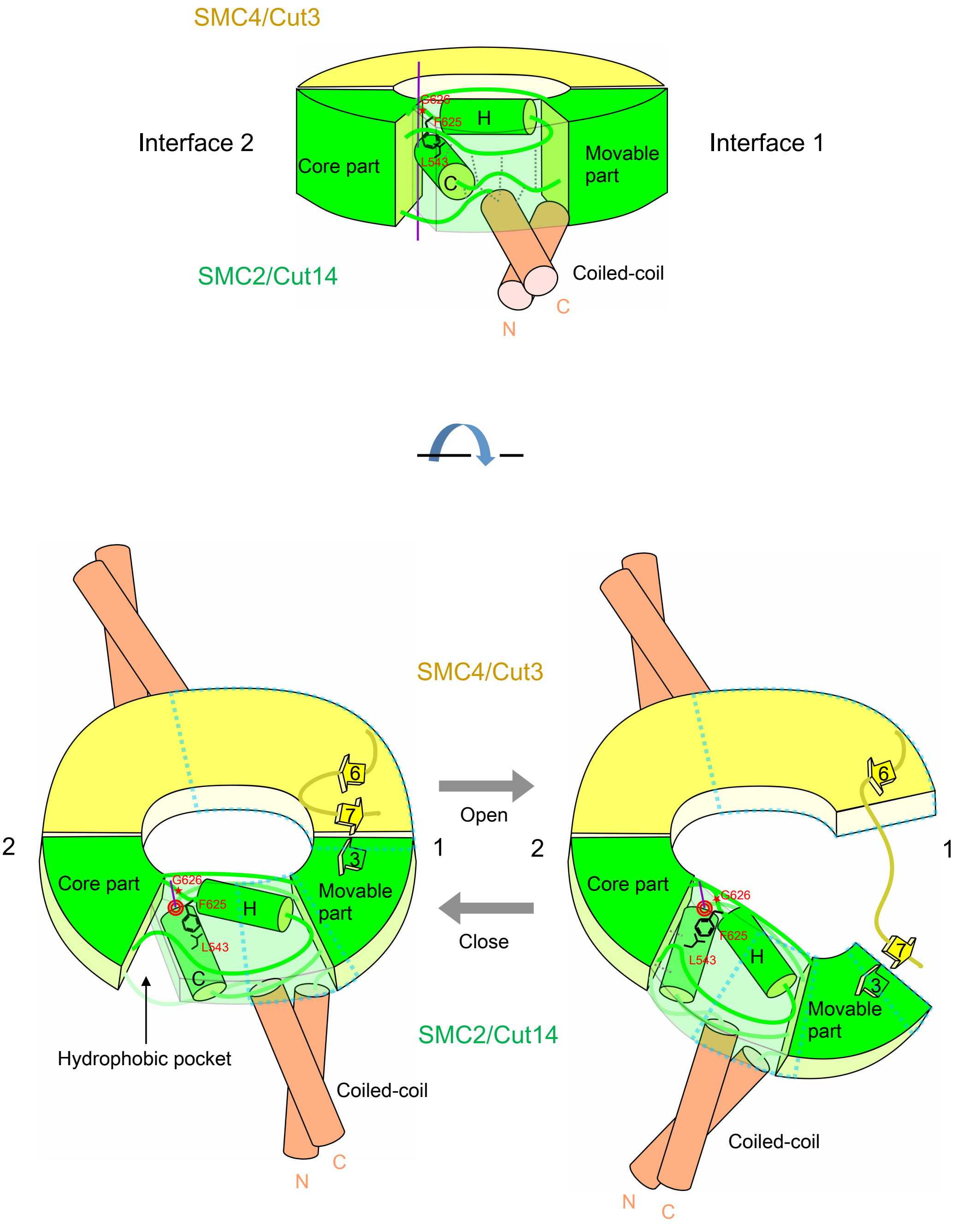

Supplemental Figure s7.
Hypothetical hinge opening is depicted. Green, SMC2/Cut14; Yellow, SMC4/Cut3. The hinge of SMC2/Cut14 consists of core, central, and movable parts. The central part contains the pivot residue G626 for the movement and the L543 mutation site (see text), and associates with the coiled coil. The hydrophobic pocket present between the central and the core disappears upon hinge opening. The movable part containing β3 associates with β7 in SMC4/Cut3 upon hinge opening so that β7 and β6 are widely separated. During hinge opening, the pivot residue keeps the same position in the hinge.

Supplemental Table s1

Cut3/SMC4 phospho- (blue residues) or thiophospho- (red residues) peptides detected by LC-MS

Specimens and peptides	Ion score and peptide amino acid sequence	
1 **None**		
770-792	4.47	WRVVTLSGQLIDKSGTMTGGGTR
793-816	2.70	VKKGGMSSAITSDVSPASVETCDK
795-824	6.68	KGGMSSAITSDVSPASVETCDKQVQLEDTR
796-816	1.86	GGMSSAITSDVSPASVETCDK
2 **+ATP**		
626-634	4.20	ASLSSSRSR
698-710	15.5	SNNLGRASFIILK
772-782	1.43	VVTLSGQLIDK
783-795	4.30	SGTMTGGGTRVKK
783-794	10.00	SGTMTGGGTRVK
1171-1186	6.39	LDEFMYGFGIISMKLK
3 **+ATPrS**		
454-463	5.60	KSIEALSFEK
621-634	11.9	LEEMKASLSSSRSR
704-710	2.77	ASFIILK
770-792	13.08	WRVVTLSGQLIDKSGTMTGGGTR
783-792	2.59	SGTMTGGGTR
783-795	16.53	SGTMTGGGTRVKK
783-795	11.08	SGTMTGGGTRVKK
793-816	8.44	VKKGGMSSAITSDVSPASVETCDK
795-816	7.57	KGGMSSAITSDVSPASVETCDK
796-816	18.49	GGMSSAITSDVSPASVETCDK
892-903	9.70	RNLQNKISNMDK
1109-1120	7.56	SELVSNISVLKK
1109-1120	7.29	SELVSNISVLKK

Supplemental Table s2

The number of Cut3/SMC4 peptides obtained after Lyc-C proteinase digestion. Only peptides containing the sequence 783-792 SGTMTGGGTR are shown.

Specimens and peptides	Peptide amino acid sequence (Phospho., Thiophospho.)	No. of peptides	Phospho. Sites (Thiophospho.)
1 None (Lys-C)			
783-794	**K.SGTMTGGGTRVK.K**	**16**	
783-794	K.SGTMTGGGTRVK.K	4	S783, T785, T787, T791
783-795	K.SGTMTGGGTRVKK.G	1	T785, T787
783-795	K.SGTMTGGGTRVKK.G	1	S783, T785, T787
783-816	K.SGTMTGGGTRVKKGGMSSAITSDV SPASVETCDK.Q	1	T785, T787, T791, S799, S800, T803, S804, S807
2 +ATP (Lys-C)			
783-794	**K.SGTMTGGGTRVK.K**	**32**	
768-795	K.TRWRVVTLSGQLIDKSGTMTGGGT RVKK.G	1	T774
783-795	K.SGTMTGGGTRVKK.G	1	T791
783-795	K.SGTMTGGGTRVKK.G	1	T787
783-795	K.SGTMTGGGTRVKK.G	1	S783, T785
783-795	K.SGTMTGGGTRVKK.G	2	T785, T791
783-795	K.SGTMTGGGTRVKK.G	2	S783, T785, T787, T791
783-816	K.SGTMTGGGTRVKKGGMSSAITSDV SPASVETCDK.Q	1	T785, T787, T791
3 +ATPγS (Lys-C)			
783-794	**K.SGTMTGGGTRVK.K**	**19**	
768-794	K.TRWRVVTLSGQLIDKSGTMTGGGT RVK.K	1	T768, T774, S783, T791
783-794	K.SGTMTGGGTRVK.K	1	T791
783-794	K.SGTMTGGGTRVK.K	1	S783, T785, T787
783-794	K.SGTMTGGGTRVK.K	1	S783, T787, T791
783-794	K.SGTMTGGGTRVK.K	1	S783, T785, T791
783-794	K.SGTMTGGGTRVK.K	2	T785, T787, T791
783-794	K.SGTMTGGGTRVK.K	1	S783, T785, T787, T791
783-795	K.SGTMTGGGTRVKK.G	1	T785, T791
783-795	K.SGTMTGGGTRVKK.G	1	T785, T787
783-795	K.SGTMTGGGTRVKK.G	1	S783, T785, T787, T791
